# Longitudinal effects of a common *UMOD* variant on kidney function, blood pressure, cognitive and physical function in older women and men

**DOI:** 10.1038/s41371-022-00781-y

**Published:** 2022-11-28

**Authors:** Engi Abdel–Hady Algharably, Linda Elizabeth Villagomez Fuentes, Sarah Toepfer, Maximilian König, Vera Regitz-Zagrosek, Lars Bertram, Juliane Bolbrinker, Ilja Demuth, Reinhold Kreutz

**Affiliations:** 1 Charité – Universitätsmedizin Berlin, corporate member of Freie Universität Berlin and Humboldt-Universität zu Berlin, Institute of Clinical Pharmacology and Toxicology, Charitéplatz 1, 10117 Berlin, Germany; 2 Charité – Universitätsmedizin Berlin, corporate member of Freie Universität Berlin and Humboldt-Universität zu Berlin, Lipid Clinic at the Interdisciplinary Metabolism Center, Berlin, Germany; 3 Charité – Universitätsmedizin Berlin, Corporate Member of Freie Universität Berlin, Humboldt-Universität zu Berlin, Institute for Gender in Medicine, Center for Cardiovascular Research, 13347 Berlin, Germany; 4 Department of Cardiology, University Hospital Zürich, University of Zürich, Zürich, Switzerland; 5 Lübeck Interdisciplinary Platform for Genome Analytics, Institutes of Neurogenetics and Cardiogenetics, University of Lübeck, Lübeck, Germany; 6 Center for Lifespan Changes in Brain and Cognition, Department of Psychology, University of Oslo, Oslo, Norway; 7 Charité – Universitätsmedizin Berlin, Berlin Institute of Health Center for Regenerative Therapies, 13353 Berlin, Germany

**Keywords:** Hypertension, Prognosis, Ageing

## Abstract

Genetic variants in *UMOD* associate with kidney function and hypertension. These phenotypes are also linked to sex-related differences and impairment in cognitive and physical function in older age. Here we evaluate longitudinal associations between a common *UMOD* rs4293393-A>G variant and changes in estimated glomerular filtration rate (eGFR), blood pressure (BP), cognitive and physical function parameters in older participants in the BASE-II after long-term follow-up as part of the GendAge study. Overall, 1010 older participants (mean age 75.7 ± 3.7 years, 51.6% women) were analyzed after follow-up (mean 7.4 years) both in cross-sectional analysis and in longitudinal analysis as compared to baseline. In cross-sectional analysis, heterozygous G–allele carriers exhibited significantly higher eGFR values (AA, 71.3 ml/min/1.73 m^2^, 95% CI, 70.3–72.3 vs. AG, 73.5 ml/min/1.73 m^2^, 95% CI, 72.1–74.9, *P* = 0.033). Male heterozygous G-allele carriers had lower odds of eGFR < 60 mL/min/1.73 m^2^ (OR 0.51, 95% CI, 0.28–0.95, *P* = 0.032) and in Timed Up and Go-Test ≥ 10 s (OR 0.50, 95% CI, 0.29–0.85, *P* = 0.011) whereas women were less likely to have hypertension (OR 0.58, CI, 0.37–0.91, *P* = 0.018). *UMOD* genotypes were not significantly associated with longitudinal changes in any investigated phenotype. Thus, while the impact of *UMOD* rs4293393 on kidney function is maintained in aging individuals, this variant has overall no impact on longitudinal changes in BP, kidney, cognitive or functional phenotypes. However, our results suggest a possible sex-specific modifying effect of *UMOD* on eGFR and physical function in men and hypertension prevalence in women.

## Introduction

Uromodulin, also named Tamm Horsfall is a 95 kDa glycoprotein and is exclusively synthesized by the thick ascending limb and to limited extent by the early distal convoluted tubule in the kidney [1]. Uromodulin plays an important role in the regulation of renal tubule salt handling presumably through modulation of the apical Na-K-2Cl cotransporter (NKCC2) transporter activity and has been linked to both blood pressure (BP) regulation as well as kidney function. Genetic polymorphisms in the *UMOD* gene identified by genome-wide association studies [2, 3] and animal models [4] have been consistently shown to associate with kidney function [5, 6], risk of chronic kidney disease (CKD) [7] but also hypertension [8]. Uromodulin has even been proposed to be a possible target for BP control and a causal relationship between uromodulin and BP was recently suggested by mendelian randomization [9]. Both hypertension and CKD have been linked to age-related impairment in cognitive and physical function [10, 11].

Recently, we reported on a cross-sectional analysis of a *UMOD* variant, a single nucleotide polymorphism rs4293393-A>G, in the noncoding region of the *UMOD* gene with BP, kidney function, as well as cognitive and physical function in 1556 participants of a community-based cohort: the Berlin Aging Study II [6]. Our original hypothesis was that *UMOD* variants associated with lower BP and/or improved kidney function in midlife could have a positive impact on cognitive and physical abilities in older age. We confirmed the association of the minor G-allele with a higher eGFR and a lower risk of eGFR <60 mL/min/1.73 m^2^ in older participants of the cohort. Nevertheless, we could not find evidence for cross-sectional associations with BP, parameters of cognition or functionality in this previous analysis at baseline. Older participants of the study exhibited a good overall health status with only relatively low prevalence of chronic disease which might partly explain the negative findings.

Sex differences in the incidence, progression as well as disease outcomes of hypertension and CKD have been recognized [12, 13]. Moreover, the rate of cognitive decline with aging is also different between men and women who are impacted differently by ageing [14]. However, little information exists about the influence of genetic components on this variation. In addition, variations in serum as well urinary uromodulin levels have been reported among women and men that might imply the presence of sex-specific differences in the regulation of uromodulin [15, 16]. Data on the effect of sex on the association of *UMOD* with BP, kidney function and parameters of cognitive and physical performance are still lacking. Here, we aim to assess in a longitudinal analysis the relationship between the *UMOD* rs4293393-A>G variant and eGFR decline, BP changes as well as the decline in parameters of cognitive and physical function in older participants of BASE-II after a mean follow-up of 7.4 years. Furthermore, the cross-sectional relationship between rs4293393 and kidney function, BP, and functional as well as cognitive assessments will be analyzed at follow-up. The evaluation will be also stratified by gender to further investigate the interaction between sex and the *UMOD* genotypes on the studied traits.

## Methods

### Participants

Participants of the older BASE-II subsample (aged ≥ 60 years) of the multidisciplinary BASE-II that took place between 2009 to 2014 in Berlin, Germany [17] were medically reassessed later in the GendAge study. The GendAge study, as a multidisciplinary and multi–institutional project, was established to examine major risk factors for cardiovascular and metabolic diseases in older adults as well as the development of major outcomes from intermediate phenotypes in the context of biological sex and gender differences. A total of 1083 participants (mean age 75.6 ± 3.8 years) with data available at baseline were investigated in the follow-up between June 2018 and March 2020 as part of GendAge study with an average follow-up of 7.4 ± 1.5 years. Details of both BASE-II and the GendAge study design have been described elsewhere [17, 18]. Genetic data of 1010 participants were available for the selected SNP, *UMOD* rs4293393, and were included in the current analysis. Genotypes were derived from Affymetrix SNP Array 6.0 analysis [19].

The study was approved by the local Ethics Committee of the Charité–Universitätsmedizin Berlin (EA2/029/09 and EA2/144/16) and all participants gave written informed consent.

### Phenotypes

#### Kidney function

Kidney function was assessed at baseline and follow-up using the creatinine-based CKD Epidemiology Collaboration (CKD–EPI) eGFR formula [20]. Study participants were stratified into categories according to their eGFR values as follows: eGFR >90 mL/min/1.73 m^2^, eGFR 60–89 mL/min/1.73 m^2^, eGFR 30–59 mL/min/1.73 m^2^, eGFR 15–30 mL/min/1.73 m^2^ and eGFR < 15 mL/min/1.73 m^2^.

#### BP measurements and definition of hypertension

Repeated attended automated BP measurements were performed at follow-up in the seated position according to a standard protocol using a validated electronic device (OMRON 500, OMRON Healthcare, Hoofddorp, Holland). For each participant, three BP measurements for systolic BP (SBP) and diastolic BP (DBP) were performed on the right and on the left arm. Mean values were used for statistical analysis and subjects with incomplete SBP and DBP measurements were excluded. Hypertension was defined as SBP ≥ 140 mmHg and/or DBP ≥ 90 mmHg [21] and/or antihypertensive treatment (self-reported or documented).

#### Assessment of cognitive function, muscle strength, and mobility

Consistent with baseline measurements, assessments of cognitive function and physical functions were repeated at follow-up. For cognitive function, we used the Mini–Mental State Examination (MMSE) and the Digit Symbol Substitution Test (DSST). The MMSE served as a tool for screening global cognitive function, with a maximum score of 30 and a cut-off score value < 24 to indicate cognitive impairment [22]. For the DSST as part of the revised Wechsler adult intelligence scale [23], which tests mainly processing speed, participants were asked to translate numbers into symbols using a key. The number of correct translations from numbers to symbols within 90 s represents the test result [23]. Similarly, physical function assessments were repeated at follow-up and included measurement of the Handgrip Strength (HGS, Scandidact, Denmark) and the Timed Up and Go–Test (TUG). For HGS, the maximum of three measurements performed for each hand was used for the analyses while the TUG was used to assess mobility by measuring the time in seconds for performing gait parameters (stand up, walk, turn, sit down) [24]. A time of 10 s was set as a cut–off value to indicate normal (<10 s) vs. impaired (≥10 s) gait performance [25].

#### Frailty and morbidity

Similar to baseline, frailty was measured using a slightly adapted version of Fried’s 5-point frailty score based on five criteria: unintentional weight loss, self-reported exhaustion, weakness, slow walking speed, and low physical activity [26, 27]. Participants were classified as frail (3–5 criteria met), pre-frail (1–2 met), or not frail (no criterion met). For statistical analysis, frail and pre-frail were grouped together. The morbidity index (MI) was computed as a modified version [28] of the Charlson index (CCI) [29]. For statistical analysis, scores ≥ 1 were combined into one variable.

### Statistical analyses

Continuous data were presented as mean and standard deviation (SD), while categorical data were presented as numbers and percentages. Genotype-phenotype associations were analyzed cross-sectionally for the follow-up data using analysis of co-variance (ANCOVA) models for continuous variables with fixed factor genotype and adjustment for sex as covariate in the overall analysis or binary logistic regression for categorical variables controlling for sex to calculate odds ratios (OR). Levene’s test for equality of variances was performed and the results indicated equal variance across compared groups. Cross-sectional analysis results are reported as mean and 95% confidence intervals (CI).

Longitudinal analyses were performed to examine genotype association with the change in the outcomes of interest over time and were controlled for sex and follow-up time in the overall analysis. Continuous variables were analyzed using ANCOVA (additionally adjusting for phenotypes’ baseline values) while Cox proportional hazards regression was used to analyze genotypes’ associations with changes observed in the categorical outcomes not present at baseline after follow-up and were recorded as incidence of new events.

Both cross-sectional and longitudinal analyses were repeated without adjusting for sex and results were sex-stratified. Moreover, for sex-specific analysis, we examined the interaction between genotype and sex and introduced the interaction term between genotype and sex as fixed effect item into the ANCOVA, binary logistic and Cox hazard regression models.

In both binary logistic and Cox hazard regression models, AG and GG genotypes were compared to AA genotype as the indicator genotype.

An available case analysis (pairwise deletion) was used, thus, participants were excluded only from analysis when data were incomplete regarding one or more variables that were relevant to the conducted analyses. *P* values < 0.05 were considered statistically significant (without adjustment for the number of tests performed). All statistical tests were two-sided and analyses were performed using SPSS 25 (SPSS Statistics Software, Armonk, NY: IBM Corp).

## Results

### Characteristics of the study population at follow-up

A total of 1010 individuals for whom genotyping data was available were studied at follow-up on average 7.4 ± 1.4 years (range 4.0–10.4 years) after baseline assessment. The mean age at follow-up was 75.7 ± 3.7 years with men and women almost equally distributed (51.6% women). Participant characteristics at baseline and follow-up are summarized in Table 1. A higher proportion of individuals were characterized as pre–frail or frail compared to baseline (53.6% vs. 32%, respectively) and 62.5% had a MI ≥ 1. As expected, mean eGFR decreased from 77.1 ± 12.1 to 72 ± 12.7 ml/min/1.73 m^2^ at follow-up and the number of subjects with eGFR<60 mL/min/1.73 m^2^ rose from 139 to 183 subjects. The change of eGFR category distribution in participants at baseline and follow-up is shown in Supplementary Fig. 1 demonstrating a higher proportion of individuals progressing from higher to lower eGFR categories, however, with no new cases of end stage kidney disease and only one participant with an eGFR < 15 mL/min/1.73 m^2^ at baseline and follow-up. On the other hand, mean follow-up SBP and DBP in the seated position were 136.4 ± 17.8 mmHg and 79.5 ± 9.9 mmHg, respectively, which were lower than the mean values obtained at baseline assessment. In total 767 participant (76%) had hypertension compared to 72.7 % at baseline. Except for MMSE which remained relatively stable, average values for functional and physical parameters assessments declined at follow-up (Table 1).

**Table 1:** Characteristics of the study population at baseline and follow-up.

Parameter	Baseline (2009–2014)		Follow-up (2018–2020)	
	*N*	Value	*N*	Value
Age (years)	1556	68.2 ± 3.7 (range 60–84)	1010	75.7 ± 3.7 (range 64–94)
<80		1546 (99.4%)		897 (88.9%)
≥80		10 (0.6%)		112 (11.1%)
Men		760 (48.8 %)		489 (48.4%)
Women		796 (51.2 %)		521 (51.6%)
BMI (kg/m^2^)	1530	26.8 ± 4.2	1008	27.0 ± 4.3
Morbidity index	1419		875	
0		497 (35%)		328 (37.5%)
≥1		922 (65%)		547 (62.5%)
Frailty index	1436		999	
Not frail		976 (68%)		463 (46.3%)
Prefrail		448 (31.2%)		491 (49.1%)
Frail		12 (0.8%)		45 (4.5%)
SCr (mg/dl)	1528	0.90 ± 0.2	1004	0.92 ± 0.27
eGFR (mL/min/1.73 m^2^)	1528	77.1 ± 12	1004	72.1 ± 12.9
eGFR <60 mL/min/1.73 m^2^		139 (9.1%)		183 (18.2%)
BP (mmHg)	1529		1008	
SBP		143.7 ± 18.7		136.4 ± 17.8
DBP		83.1 ± 10.9		79.53 ± 9.9
Hypertension	1529	1112 (72.7%)	1009	767 (76%)
With antihypertensives treatment		592 (53.2%)		576 (57.1%)
Thereof controlled				
BP < 140/90 mmHg		220 (37.2%)		327 (32.4%)
BP < 140/80 mmHg		153 (25.8%)		245 (24.3%)
MMSE	1534	28.5 ± 1.6	998	28.5 ± 1.5
DSST	1339	44.6 ± 8.5	1005	40.7 ± 8.8
TUG (s)	1531	7.9 ± 1.9	1005	8.7 ± 2.6
HGS (kg)	1532	34.2 ± 9.7	1008	27.4 ± 9.2

Data are given as mean ± standard deviation or as numbers and percentages in parentheses.

*BMI* body mass index, *SCr* serum creatinine, *eGFR* estimated glomerular filtration rate according to CKD–EPI creatinine equation, *BP* blood pressure, *SBP* systolic BP, *DBP* diastolic BP, *MMSE* Mini–Mental State Examination, *DSST* Digit Symbol Substitution Test, *TUG* Timed Up and Go-Test, *HGS* Handgrip Strength.

### Cross-sectional analysis for the association between UMOD rs4293393 and phenotypes at follow-up

Genotype distribution for *UMOD* rs4293393 at follow-up (*N* = 1010) was similar to that at baseline (*N* = 1556) [6] with the genotype AA in 671 (66.4%), AG in 300 (29.7%) and GG in 39 (3.9%) participants which also shows a comparable minor allele frequency (MAF) of 18.7%.

**Fig. 1:**
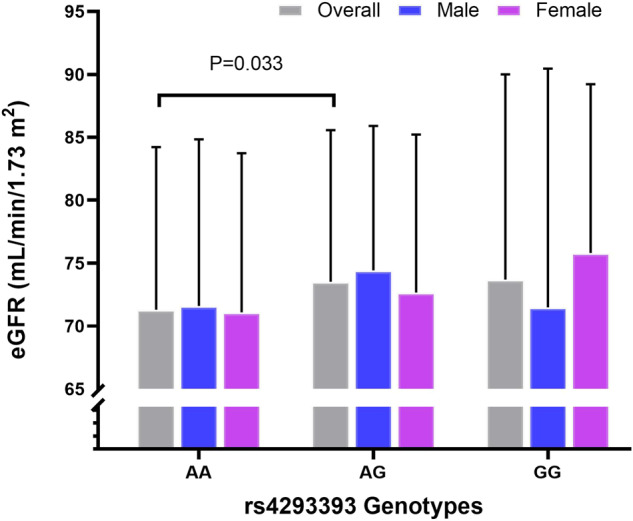
*UMOD* rs4293393 association with estimated glomerular filtration rate (eGFR): overall and sex stratified cross-sectional analysis. *P* = 0.003 (post hoc analysis) comparing mean values of heterozygous AG (*n* = 297) with homozygous AA (*n* = 668) individuals. Mean eGFR value for GG genotype was not significantly different from that of AA and AG groups. Data were analyzed by analysis of covariance, adjusted for sex in the overall analysis.

Consistent with the results at baseline, the mean eGFR was associated with *UMOD* genotypes in the overall analysis adjusted for sex only. Heterozygous carriers of the minor G-allele continued to show significantly higher eGFR values compared to individuals homozygous for the risk A-allele (AA, *n* = 668, 71.3 ml/min/1.73 m², 95% CI, 70.3–72.3 vs. AG, *n* = 297, 73.5 ml/min/1.73 m², 95% CI, 72.1–74.9 vs. GG, *n* = 39, 73.7 ml/min/1.73 m², 95% CI, 69.7–77.7, *P* = 0.033, Fig. 1, Supplementary Table 1) and to have lower odds of eGFR < 60 mL/min/1.73 m^2^ (AG, OR: 0.68, 95% CI, 0.47–0.99, *P* = 0.045; GG, OR: 1.1, 95% CI, 0.49–2.63, *P* = 0.765). On the other hand, no significant association was found between genotypes and hypertension status (AG, OR: 1.31, 95% CI, 0.95–1.82, *P* = 0.102; GG, OR: 0.88, 95% CI, 0.41–1.88, *P* = 0.765), SBP and DBP (Table 2), parameters of cognitive or physical function (Tables 3 and 4).

**Table 2:** Overall and sex-stratified association of rs4293393 with BP (cross-sectional analysis) and BP changes (longitudinal analysis).

Phenotype	*UMOD* rs4293393			*P*
	AA	AG	GG	
*Cross-sectional analysis*				
SBP (mmHg)	*N* = 669	*N* = 300	*N* = 39	
Overall	136.3 (134.9–137.6)	136.7 (134.7–138.7)	136.1 (130.5–141.7)	0.933^a^
Women	134.3 (132.4–136.2)	135.8 (133.0–138.6)	136.3 (128.5–144.1)	0.641
Men	138.3 (136.4–140.2)	137.7 (134.7–140.6)	135.9 (127.9–143.9)	0.813
*DBP (mmHg)*				
Overall	79.5 (78.7–80.3)	79.6 (78.5–80.8)	79.4 (76.3–82.5)	0.976^a^
Women	79.5 (78.5–80.6)	79.8 (78.3–81.4)	78.6 (74.3–82.9)	0.864
Men	79.5 (78.4–80.5)	79.4 (77.9–81.1)	80.2 (75.7–84.7)	0.947
*Longitudinal analysis*				
SBP change (mmHg)	*N* = 665	*N* = 300	*N* = 39	
Overall	−6.6 (−8.1–5.0)	−6.8 (−9.1–−4.6)	−8.6(−14.9–−2.4)	0.926^a^
Women	−8.4 (−10.1–−6.6)	−7.0 (−9.5–−4.4)	−7.1 (−14.3–0.7)	0.647
Men	−5.0 (−6.8–−3.2)	−6.12 (−8.8–−3.4)	−8.13 (−15.5–−0.8)	0.620
DBP change (mmHg)	*N* = 665	*N* = 300	*N* = 39	
Overall	−3.6 (−4.44–−2.77)	−3.5 (−4.74–−2.26)	−4.6 (−8.08–−1.18)	0.922^a^
Women	−3.4 (−4.8–−2.5)	−2.3 (−4.3–−1.5)	−3.4 (−7.3–0.5)	0.813
Men	−3.9 (−4.9–−2.9)	−4.2 (−5.7–−2.7)	−4.9 (−8.9–−0.8)	0.861

Data were analyzed by analysis of covariance and are given as means with 95% confidence intervals in brackets adjusted for sex in the overall cross-sectional analysis, for follow-up time, baseline values and sex in the overall longitudinal analysis and for follow-up time and baseline values in the sex-stratified longitudinal analysis.

*BP* blood pressure, *SBP* systolic BP, *DBP* diastolic BP.

^a^*P* > 0.05 for sex-genotype interaction.

**Table 3:** Overall and sex-stratified analysis of rs4293393 association with parameters of cognitive (MMSE, DSST) and physical function (HGS): cross-sectional and longitudinal analysis.

Phenotype	*UMOD* rs4293393			*P*
*Cross-sectional analysis*				
	AA	AG	GG	
MMSE, *n* = 998	*N* = 660	*N* = 299	*N* = 39	
Overall	28.6 (28.5–28.7)	28.5 (28.4–28.7)	28.2 (27.7–28.7)	0.298^a^
Women	28.8 (28.6–28.9)	28.7 (28.5–28.9)	28.3 (27.6–28.9)	0.345
Men	28.4 (28.2–28.6)	28.3 (28.1–28.6)	28.2 (27.5–28.9)	0.750
DSST, *n* = 1005	*N* = 668	*N* = 298	*N* = 39	
Overall	40.8 (40.1–41.5)	40.6 (39.60–41.5)	41.4 (38.7–44.2)	0.810^a^
Women	41.9 (41.0–42.9)	42.1 (40.7–43.5)	41.3 (37.5–45.2)	0.928
Men	39.6 (38.6–40.5)	38.9 (37.4–40.3)	41.6 (37.7–45.5)	0.401
HGS (kg, *n* = 1008)	*N* = 669	*N* = 300	*N* = 39	
Overall	27.4 (26.9–27.80)	27.7 (27.0–28.3)	26.1 (24.4–27.9)	0.261^a^
Women	20.5 (19.9–21.1)	20.7 (19.8–21.5)	19.2 (16.8–21.7)	0.548
Men	34.7 (34.1–35.3)	35.1 (34.2–36.1)	33.6 (31.1–35.3)	0.464
*Longitudinal analysis*				
MMSE change	*N* = 652	*N* = 295	*N* = 39	
Overall	−0.03 (−0.15–0.09)	−0.1 (−0.27–0.07)	−0.45 (−1.0–−0.03)	0.225^a^
Women	0.13 (−0.03–0.30)	0.06 (−0.18–0.30)	−0.50 (−1.15–0.18)	0.206
Men	−0.19 (−0.40–−0.02)	−0.25 (−0.50–0.00)	−0.41 (−1.09–0.28)	0.777
DSST change	*N* = 586	*N* = 260	*N* = 31	
Overall	−4.0 (−4.5–−3.5)	−4.9 (−5.7–−4.2)	−4.4 (−6.6–−2.3)	0.137^a^
Women	−3.5 (−4.2–−2.8)	−4.2 (−5.2–−3.2)	−4.0 (−6.8–−1.2)	0.514
Men	−4.6 (−5.2–−3.9)	−5.6 (−6.6–4.6)	−4.6 (−7.5–−1.6)	0.238
HGS change (kg)	*N* = 664	*N* = 297	*N* = 39	
Overall	−6.9 (−7.3–−6.6)	−7.0 (−7.5–−6.5)	−7.5 (−8.9–−6.1)	0.746^a^
Women	−9.6 (−10.2–−9.0)	−9.9 (−10.7–−9.2)	−10.5 (−12.5–−8.5)	0.623
Men	−4.1 (−4.7–−3.5)	−4.0 (−4.8–−3.1)	−4.3 (−6.4–−2.3)	0.923

Data were analyzed by analysis of covariance and are given as means with 95% confidence intervals in brackets adjusted for sex in the overall cross-sectional analysis, for follow-up time, baseline values and sex in the overall longitudinal analysis and for follow-up time and baseline values in the sex-stratified longitudinal analysis.

*MMSE* Mini–Mental State Examination, *DSST* Digit Symbol Substitution Test, *HGS* Handgrip Strength.

^a^*P* > 0.05 for sex-genotype interaction.

**Table 4:** Overall and sex-stratified analysis of rs4293393 association with TUG, frailty and morbidity index outcomes at follow-up (cross-sectional analysis).

Phenotype	*UMOD* rs4293393	
	AG	GG
TUG ≥ 10 s	OR [95% CI], *P*	
Overall	1.32 [0.93–1.89], *P* = 0.102	1.00[0.46–2.20], *P* = 0.988
Women	1.10 [0.68–1.77], *P* = 0.708	1.40 [0.47–4.11], *P* = 0.544
Men	0.50 [0.29–0.85], ***P*** = **0.011**	0.70 [0.22–2.23]; *P* = 0.545
Prefrail/frail	OR [95% CI], *P*	
Overall	1.20 [0.91–1.59], *P* = 0.174	1.18 [0.61–2.27], *P* = 0.620
Women	0.85 [0.58–1.24], *P* = 0.386	1.84 [0.69–4.93], *P* = 0.222
Men	0.81[0.55–1.21, *P* = 0.299	0.77 [0.30–1.95], *P* = 0.580
Morbidity index ≥ 1	OR [95% CI], *P*	
Overall	1.01 [0.75–1.37], *P* = 0.940	0.96 [0.47–1.96], *P* = 0.919
Women	1.24 [0.81–1.89], *P* = 0.325	0.82 [0.31–2.16], *P* = 0.692
Men	0.78 [0.51–1.21], *P* = 0.271	1.16 [0.39–3.46], *P* = 0.786

Data were analyzed by binary logistic regression and are given as odds ratios (OR) with 95% confidence intervals in brackets adjusted for sex in the overall analysis. In logistic regression models for TUG ≥ 10 s, for being prefrail or frail and for morbidity index ≥ 1, genotype was added as fixed factor and AA genotype was set as reference group.

*P* values < 0.05 were marked in bold.

For the interaction effect between sex and genotype for TUG ≥ 10 s, *P* = 0.086; for being prefrail or frail, *P* = 0.467 and for morbidity index ≥ 1, *P* = 0.257.

*TUG* Timed Up and Go-Test.

Subgroup analysis for men and women revealed no sex-specific association of *UMOD* genotypes with BP phenotypes, MMSE, DSST or HGS (Tables 2 and 3). A non-significant sex-genotype interaction was observed for eGFR (*P* = 0.390), SBP (*P* = 0.562), DBP (*P* = 0.838), MMSE (*P* = 0.310), DSST (*P* = 0.464) and HGS (*P* = 0.937). Similarly, men and women did not differ significantly in their genotype association with frailty and MI (Table 4). On the other hand, male but not female heterozygous G-allele carriers had significantly lower odds of eGFR < 60 mL/min/1.73 m^2^ (men: AG, OR: 0.51, 95% CI, 0.28–0.95, *P* = 0.032; vs. women: AG, OR: 0.83, 95% CI, 0.51–1.34, *P* = 0.444) and lower the odds for TUG ≥ 10 s (Table 4), however, with a non-significant sex-genotype interaction for eGFR <60 mL/min/1.73 m^2^ (*P* = 0.144) and TUG ≥ 10 s (*P* = 0.086). Furthermore, female heterozygous G-allele carriers had significantly lower odds of hypertension (men: AG, OR: 1.04, 95% CI, 0.64–1.69, *P* = 0.869 vs. women: AG, OR: 0.58, 95% CI, 0.37–0.91, *P* = 0.018). The corresponding sex-genotype interaction was non-significant (*P* = 0.143).

### Longitudinal analysis for the association between UMOD rs4293393 and phenotypes

**Fig. 2:**
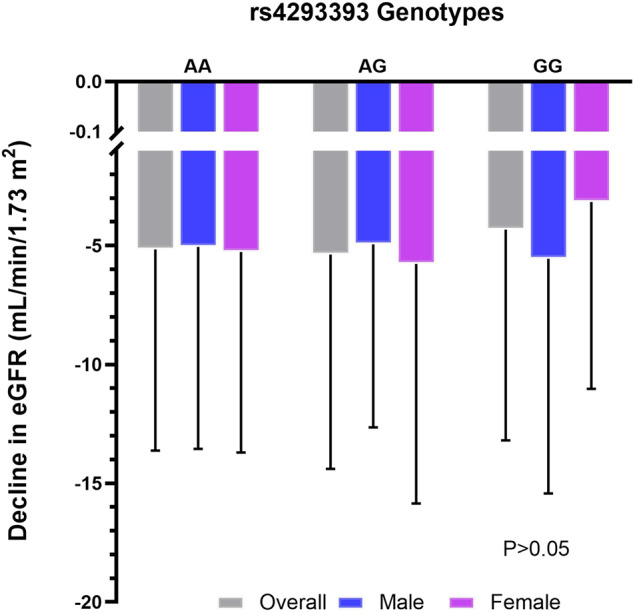
*UMOD* rs4293393 association with decline in estimated glomerular filtration rate (eGFR): overall and sex stratified longitudinal analysis. *P* > 0.05 across all groups. Data were analyzed by analysis of covariance, adjusted for sex, follow-up time and baseline eGFR values in the overall analysis and for follow-up time and baseline values eGFR in the sex-subgroup analysis.

Although homozygous G-allele carriers displayed the lowest decline in eGFR after a mean follow-up of 7.4 years, the association was not statistically significant (AA, −5.3 ml/min/1.73 m², 95% CI, −5.9–4.6 vs. AG, −5.1 ml/min/1.73 m², 95% CI, −6.1–4.2 vs. GG, −4.2 ml/min/1.73 m², 95% CI, −6.8–1.6; *P* = 0.708, Fig. 2, Supplementary Table 1).

We examined the association of rs4293393 genotypes with the decline in kidney function after follow-up in terms of incidence of new cases of eGFR decline i.e., from eGFR ≥ 60 ml/min/1.73 m² to eGFR < 60 ml/min/1.73 m² or decline from a higher eGFR category at baseline to lower category at follow-up by means of Cox hazard regression. In the overall analysis, homozygous and heterozygous G-allele carriers, though exhibiting numerically lower hazard ratios (HRs) for an eGFR < 60 mL/min/1.73 m^2^ compared to homozygous risk A-allele carriers, the association was not statistically significant (AG, HR: 0.83, 95% CI, 0.55–1.51, *P* = 0.380 vs. GG, HR: 0.55, 95% CI, 0.17–1.74, *P* = 0.306). Similar results were observed for eGFR category decline (AG, HR: 0.99, 95% CI, 0.75–1.32; *P* = 0.960 vs. GG, HR: 0.87, 95% CI, 0.44–1.69; *P* = 0.672).

We found no significant association with longitudinal BP changes using SBP, DBP (Table 2) or the risk of incident hypertension at follow-up (*P* = 0.949). Similarly, no association was found with longitudinal changes in MMSE, DSST, HGS (Table 3) or in participants’ frailty, TUG and MI status after follow-up (Table 5).

**Table 5:** Overall and sex-stratified analysis of rs4293393 association with changes in TUG, frailty and morbidity index outcomes (longitudinal analysis).

Phenotype	*UMOD* rs4293393	
	AG	GG
TUG ≥ 10 s	HR [95% CI], *P*	
Overall	0.82 [0.57–1.17], *P* = 0.271	0.89 [0.42–1.92], *P* = 0.774
Women	0.92 [0.56–1.50], *P* = 0.735	1.09 [0.39–3.02], *P* = 0.874
Men	0.71 [0.42–1.20], *P* = 0.203	0.71 [0.22–2.27], *P* = 0.564
Prefrail/frail	HR [95% CI], *P*	
Overall	0.95 [0.75–1.22], *P* = 0.700	1.15[0.69–1.92], *P* = 0.587
Women	1.00 [0.70–1.42], *P* = 0.556	1.27 [0.66–2.44], *P* = 0.480
Men	1.00 [0.70–1.42], *P* = 0.995	0.97 [0.42–2.21], *P* = 0.940
Morbidity index ≥ 1	HR [95% CI], *P*	
Overall	1.05 [0.72–1.54], *P* = 0.798	1.88 [0.80–4.69], *P* = 0.175
Women	1.48 [0.87–2.53], *P* = 0.149	2.39 [0.73–7.91], *P* = 0.152
Men	0.78 [0.44–1.36], *P* = 0.372	1.52 [0.36–6.35], *P* = 0.567

Data were analyzed by Cox regression and are given as hazard ratios (HR) with 95% confidence intervals in brackets adjusted for sex and follow-up time in the overall analysis and for follow-up time in the sex sub-group analysis. In Cox regression models for new events of TUG ≥ 10 s, being prefrail or frail and morbidity index ≥ 1, genotype was added as fixed factor and AA genotype was set as reference group.

For the interaction effect between sex and genotype for TUG ≥ 10 s, *P* = 0.678; for being prefrail or frail, *P* = 0.724 and for morbidity index ≥ 1, *P* = 0.252.

*TUG* Timed Up and Go-Test.

Sex-stratified analysis yielded no significant association with longitudinal changes in eGFR with a non-significant sex-genotype interaction (*P* = 0.582). Similarly, men and women did not differ significantly regarding changes in SBP and DBP (Table 2), risk of incident hypertension (*P* = 0.894), risk of eGFR < 60 mL/min/1.73 m^2^ (men, *P* = 0.644; women, *P* = 0.446), risk of eGFR category decline (men, *P* = 0.878; women, *P* = 0.653), decline in cognitive and physical function assessment (Tables 3 and 5).

## Discussion

We have previously reported on the association of rs4293393 in the promotor of the *UMOD* gene with kidney function-related traits but not with BP-, cognitive- or physical function-related traits in a cohort of community-dwelling older individuals [6]. In this study, we utilize follow-up data from older adults of the BASE-II cohort to evaluate the genetic association of the same *UMOD* variant with longitudinal changes in the same outcomes after up to 10 years follow-up and whether different effects could be discerned for aging men and women.

We also repeated the previously reported cross-sectional analyses using data from the follow-up examinations.

In this cohort study, the association of the minor G-allele of rs4293393 with kidney function-related traits after follow-up was replicated as reported earlier for the baseline cohort [6]. Heterozygous G-allele carriers retained higher levels of eGFR and lower odds of impaired kidney function i.e., eGFR < 60 mL/min/1.73 m^2^ with men showing significantly lower odds than women. Therefore, kidney function outcomes show correlation between baseline and follow-up data.

We found no association between the rs4293393-genotypes and BP phenotypes in either the cross-sectional analysis of the follow-up data or the longitudinal analysis. Men and women did not differ significantly with respect to their genotypic association with BP values although heterozygous G-allele carriers among women were less likely to have hypertension compared to men. Sex differences in BP regulation and susceptibility to hypertension have been long identified and epidemiological studies indicate that men have higher BP than age-matched, premenopausal women counterparts [30]. While BP is typically lower in premenopausal women than in age-matched men, the prevalence of hypertension in women rises after menopause [30]. Given our analyses was based on the older cohort of the BASE-II (mean age 68.2 ± 3.7 years at inclusion and 75.7 ± 3.7 years at follow-up), a female population of postmenopausal women was studied. Despite the lack of a significant association with BP values, an association was detected with prevalence of hypertension after follow-up only in women. This suggests that the *UMOD-* rs4293393 variant might play a role in postmenopausal women modifying their aging pattern regarding prevalent hypertension. This relationship was not obtained for the whole cohort and was not confirmed by longitudinal subgroup analysis. A previous study in a population-based Japanese cohort (mean age 66.4 ± 11 and 63.3 ± 11 for men and women, respectively) also found a positive association between another *UMOD* variant, rs6497476, and hypertension status but not with BP levels [31]. However, in that study patients were not treated with antihypertensive medications [31]. In a family-based cohort of Han Chinese people, rs4293393 was significantly associated only with pulse pressure response to a low-salt diet intervention [32]. Notably, in our study the mean SBP and DBP values were lower at follow-up compared to baseline and the rates of BP control were nearly similar for the baseline and the follow-up cohort (see Table 1). The latter was mainly treated with antihypertensive medications with a higher proportion compared to baseline (57.1% vs. 53.2%, respectively).

Antihypertensive medications could exert BP and renoprotective effects which reflect not only on BP control but also a preserved kidney function. The impact of BP-lowering treatment and adherence to regimens on BP outcomes is challenging for longitudinal studies as well as for cross-sectional BP studies [33]. Furthermore, dietary factors such as salt intake, shown to affect the expression of NKCC2, may influence BP response [4]. Taken together, longitudinal changes in BP in this population might not be sufficient to uncover a possible genetic association of the *UMOD* polymorphism with BP and/or hypertension status.

The kidney function of the cohort at follow-up was also preserved with a mean eGFR decline of only −5.0 mL/min/1.73 m^2^ over a mean follow-up period of 7.4 years. Given the mean annual decline of GFR is approximately −1.0 mL/min/1.73 m^2^ per year in elderly subjects (−0.72 and −0.92 ml/min/1.73 m^2^ per year in healthy aging men and women, respectively) [34], kidney function of the studied cohort demonstrates modest functional impairment. The distribution of eGFR categories shows that about 10% more individuals had eGFR in 30–59 mL/min/1.73 m^2^ group at follow-up compared to baseline, with no new cases of end stage kidney disease. The percentage of individuals estimated to have CKD based on eGFR_crea_ criteria alone and a single measurement of serum creatinine was doubled at follow-up but only amounted to 18.2% of the whole cohort. Lower degrees of impairment observed after follow-up might contribute to the negative results obtained for the longitudinal association with kidney function.

Formerly, we hypothesized that the protective effects exerted by the G-allele on BP and kidney function might extend to influence the physical and mental performance of individuals. However, we could not find statistical evidence of a relationship between rs4293393-genotypes with the applied tests for cognitive assessment or for physical function in the overall cross-sectional or longitudinal analyses. In view of the good BP control profiles and well-preserved kidney function of the cohort, an effect transcending to cognitive and physical performance might be less likely to spot. Besides, descriptive data of the cohort indicate an almost unchanged mean MMSE score despite a lower DSST score (average decrease ~4 points) after follow-up. However, this should be interpreted cautiously, since MMSE is a global index of cognitive performance and is sensitive for confounders such as age and education [35]. According to a recent systematic review, MMSE use should be restricted to higher age categories since the highest annual decline was found to be between ages 84–105 years [35].

Age-related diseases and conditions, physical function and frailty are prone to sexual dimorphism where women are more likely to be frailer with aging and men still perform better in physical function examinations [36]. In this study, we could not find sex-specific differences in physical and cognitive parameters except for TUG in the cross-sectional analysis at follow-up. Only male heterozygous G-allele carriers had significantly lower odds for having TUG ≥ 10 s suggesting a possible sex-specific role of this *UMOD* variant in the aging-related traits. This finding was, however, not replicated in the longitudinal analysis.

Limited data exist on the longitudinal association of *UMOD* variants with kidney function. The recently published C-STRIDE study [37] in CKD stage 1–4 patients (*n* = 2731, 40% women) of Chinese ethnicity, found that rs4293393 genotypes were associated with the risk of all-cause mortality but not the level of serum uromodulin, slope of eGFR decline or risk of cardiovascular disease events [37]. Likewise, the authors could not find a significant association with BP. Notably, the majority of their population (89.5%) were less than 67 years, and the follow-up duration was only 4.9 years. Moreover, rs4293393 had a lower MAF in east Asians compared to European populations.

Plausible reasons for the negative findings in our longitudinal analyses could be the small changes observed in the analyzed variables and the fact that the studied cohort is too healthy compared to the German norm as previously described [17]. The BASE-II cohort is reportedly a healthy cohort with low prevalence of comorbidities as well as low percentage of participants classified as frail/prefrail. Overall, 28.5% of participants at baseline (mean age 68.3 years) and 53.5% at follow-up (mean age 75.6 years) are classified as frail/prefrail by the Fried criteria. This percentage is lower than those reported in comparable cohorts. Ahrenfeldt et al. [38] reported 54.6% in the SHARE study (mean age, 66.2 years; 45.7% women) to be frail according to the Fried criteria. Similarly, Fried et al.[26] found 48% (age: 65–74 years) to be frail or prefrail. Furthermore, the BASE-II participants have a higher educational status compared to the general German population [17]. The BASE-II was recruited as a convenience sample from Berlin, an urban area with high socioeconomic levels, improved health care systems, and increased health awareness [17].

An association of *UMOD*-rs4293393 with the study’s outcomes could not be altogether discounted but might be attenuated due to the limitations of the study. The latter include the limited number of assessments obtained during follow-up. In all performed longitudinal assessments, the method of inferring the decline or change in function relied on two measurements. Multiple-occasion testing enables the evaluation of trait trajectories overtime which could help better understand the progression of diseases utilizing full trajectory of longitudinal outcomes. In addition, small sample size especially after follow-up could limit the power to detect subtle effects exerted by the *UMOD* variant in the context of aging. Our results may not be generalizable to other populations because of the sociodemographic limitations as well as the lack of ethnic diversity. We also have to mention that our study is explorative in nature and that we have not corrected our results for multiple testing. Thus, further studies verifying our results are recommended.

Strengths of the current study include the longitudinal design with a relatively long follow-up duration and a well-characterized population-based cohort, besides, balanced men to women distribution that reduces sex bias and enables exploration of sex-related differences. To our knowledge, a longitudinal genotype-phenotype association of *UMOD* variants depending on sex has not been described.

In conclusion, we extend upon our previous findings that the *UMOD* rs4293393 minor allele continues to be positively associated with kidney function in an aging population-based cohort. Although we could not find significant associations with BP phenotypes, cognitive and physical function assessments in the longitudinal analysis, our cross-sectional analysis suggests sexual disparities in the association with the risk of hypertension, impaired kidney function as well as physical function.

### Summary

#### What is known about the topic

- Genetic variants in the *UMOD* gene have been associated with kidney function, risk of chronic kidney disease and hypertension with evidence coming mostly from cross-sectional studies. These variables are subject to sex-related differences and have been linked to cognitive and physical function impairment in older adults.
- Favorable effects of *UMOD* on kidney function and/or blood pressure might reflect beneficially on the cognitive and physical functionality and modify the aging pattern in older women and men.

#### What this study adds

- We extend and replicate our previous findings on the consistent association between the minor allele of common *UMOD* variant and better kidney function in a population-based cohort of older adults after long term follow-up.
- Although no association with blood pressure, or parameters of cognitive and physical function was detected in the longitudinal analyses, cross-sectional analyses of data revealed sex-related differences in the risk of hypertension, eGFR < 60 mL/min/1.73 m^2^ and in physical function between aging women and men.

## Supplementary information

Supplementary Table 1. Overall and sex-stratified association of UMOD rs4293393 with estimated glomerular filtration rate (eGFR) (cross-sectional analysis) and eGFR changes (longitudinal analysis).

Supplementary Figure 1. Distribution of eGFR categories for study individuals at baseline (N= 1,002) and after follow-up (N= 1,004).

## Data Availability

Correspondence and requests for materials should be addressed to Engi Algharably.
