# Supplementary material for: Longitudinal effects of a common UMOD variant on kidney function, blood pressure, cognitive and physical function in older women and men
Source: J Hum Hypertens. 2022 Nov 28;37(8):709–17. doi: 10.1038/s41371-022-00781-y (PMC10403350; doi:10.1038/s41371-022-00781-y)
Supplement: Supplementary file 1 — Supplementary Table 1. Overall and sex-stratified association of UMOD rs4293393 with estimated glomerular filtration rate (eGFR) (cross-sectional analysis) and eGFR changes (longitudinal analysis). [file 41371_2022_781_MOESM1_ESM.docx]

**Longitudinal effects of a common UMOD variant on kidney function, blood pressure, cognitive and physical function in older women and men**

**Supplementary Table 1. Overall and sex-stratified association of *UMOD* rs4293393 with estimated glomerular filtration rate (eGFR) (cross-sectional analysis) and eGFR changes (longitudinal analysis).**

| Phenotype | ***UMOD* Genotype** | | | P |
| --- | --- | --- | --- | --- |
|  | AA | AG | GG |  |
| **eGFR (mL/min/1.73 m^2^)** | | | | |
|  | **N=668** | **N= 297** | **N= 39** |  |
| Overall | 71.3 (CI: 70.3–72.3) | 73.5 (CI: 72.1–74.9) | 73.7 (CI: 69.7–77.7) | 0.033^a^ |
|  | N=324 | N=143 | N=19 |  |
| Men | 71.3 (69.9 ─ 72.7) | 74.09 (72.0 ─ 76.2) | 71.31 (65.6 ─ 77.1) | 0.093 |
|  | N=344 | N=154 | N=20 |  |
| Women | 71.30 (69.9 ─ 72.7) | 72.94 (70.9 ─ 75.0) | 76.0 (70.4 ─81.6) | 0.147 |
| **Change in eGFR (mL/min/1.73 m^2^)** | | | | |
|  | **N=661** | **N=296** | **N=39** |  |
| Overall | -5.3 (-5.9 ─ -4.6) | -5.1 (-6.1 ─ -4.2) | -4.2 (-6.8─ -1.6) | 0.708^a^ |
|  | N=323 | N=142 | N=19 |  |
| Men | -5.6 (-6.5 ─ -4.7) | -5.1 (-6.4─ -3.7) | -5.9 (-9.6 ─ -2.2) | 0.781 |
|  | N=338 | N=154 | N=20 |  |
| Women | -5.0 (-5.9 ─ -4.1) | -5.2 (-6.5 ─ -3.9) | -2.5 (-6.1 ─ 1.0) | 0.403 |

Data were analyzed by analysis of covariance and are given as means with 95% confidence intervals in brackets adjusted for sex in the overall cross-sectional analysis, for follow-up time, baseline eGFR values and sex in the overall longitudinal analysis and for follow-up time and baseline eGFR values in the sex-stratified longitudinal analysis.

^a^P > 0.05 for sex-genotype interaction.
