# Supplementary material for: Longitudinal effects of a common UMOD variant on kidney function, blood pressure, cognitive and physical function in older women and men
Source: J Hum Hypertens. 2022 Nov 28;37(8):709–17. doi: 10.1038/s41371-022-00781-y (PMC10403350; doi:10.1038/s41371-022-00781-y)
Supplement: Supplementary file 2 — Supplementary Figure 1. Distribution of eGFR categories for study individuals at baseline (N= 1,002) and after follow-up (N= 1,004). [file 41371_2022_781_MOESM2_ESM.docx]

“**Longitudinal effects of a common *UMOD* variant on kidney function, blood pressure, cognitive and physical function in older women and men**”

**Supplementary Figure 1**


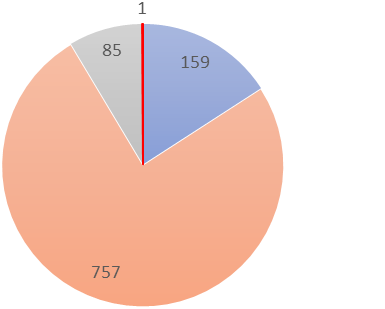

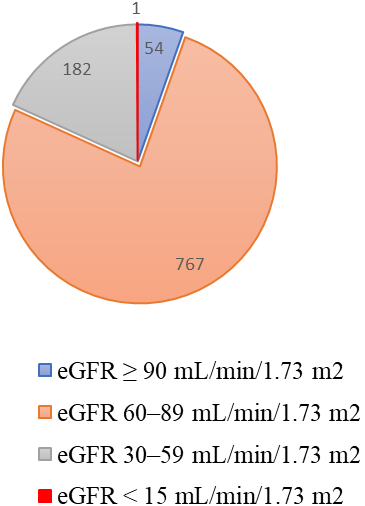


**Baseline**

**Follow-up**

**Supplementary Figure** **1**. Distribution of eGFR categories for study individuals at baseline (N= 1,002) and after follow-up (N= 1,004).

|  |  |  |  |  |  |  |  |  |  |  |  |
| --- | --- | --- | --- | --- | --- | --- | --- | --- | --- | --- | --- |
| \|  \| \| --- \| |  |  |  |  |  |  |  |  |  |  |  |
|  |  |  |  |  |  |  |  |  |  |  |  |
|  |  |  |  |  |  |  |  |  |  |  |  |
